# Construction of mRNA prognosis signature associated with differentially expressed genes in early stage of stomach adenocarcinomas based on TCGA and GEO datasets

**DOI:** 10.1186/s40001-022-00827-4

**Published:** 2022-10-17

**Authors:** Fuquan Jiang, Haiguan Lin, Hongfeng Yan, Xiaomin Sun, Jianwu Yang, Manku Dong

**Affiliations:** Department of General Surgery, PLA Strategic Support Force Characteristic Medical Center, Beijing, 100101 People’s Republic of China

**Keywords:** Data mining, Prognosis, Biological maker, Expression difference, Functional enrichment analysis

## Abstract

**Background:**

Stomach adenocarcinomas (STAD) are the most common malignancy of the human digestive system and represent the fourth leading cause of cancer-related deaths. As early-stage STAD are generally mild or asymptomatic, patients with advanced STAD have short overall survival. Early diagnosis of STAD has a considerable influence on clinical outcomes.

**Methods:**

The mRNA expression data and clinical indicators of STAD and normal tissues were obtained from The Cancer Genome Atlas (TCGA) and Gene Expression Omnibus (GEO) database. The gene expression differences were analyzed by R packages, and gene function enrichment analysis was performed. Kaplan–Meier method and univariate Cox proportional risk regression analysis were used to screen differential expressed genes (DEGs) related to survival of STAD patients. Multivariate Cox proportional risk regression analysis was used to further screen and determine the prognostic DEGs in STAD patients, and to construct a multigene prognostic prediction signature. The accuracy of predictive signature was tested by receiver operating characteristic (ROC) curve software package, and the nomogram of patients with STAD was drawn. Cox regression was used to investigate the correlation between multigene prognostic signature and clinical factors. The predictive performance of this model was compared with two other models proposed in previous studies using KM survival analysis, ROC curve analysis, Harrell consistency index and decision curve analysis (DCA). qRT-PCR and Western blot were used to verify the expression levels of prognostic genes. The pathways and functions of possible involvement of features were predicted using the GSEA method.

**Results:**

A total of 569 early-stage specific DEGs were retrieved from TCGA-STAD dataset, including 229 up-regulated genes and 340 down-regulated genes. Enrichment analysis showed that the early-stage specific DEGs were associated with cytokine–cytokine receptor interaction, neuroactive ligand–receptor interaction, and calcium signaling pathway. Multiple Cox regression algorithm was used to identify 10 early-stage specific DEGs associated with overall survival (*P* < 0.01) of STAD patients, and a multi-mRNA prognosis signature was established. The patients were divided into high-risk group and low-risk group according to the risk score. In the training set, the prognostic signature was positively correlated with tumor size and stage (*P* < 0.05), survival curve (*P* < 0.001) and time-dependent ROC (AUC = 0.625). In the training dataset and test dataset, the both signatures had good predictive efficiencies. Cox regression and DCA analysis revealed that the prognostic signature was an independent factor and had a better predict effect than the conventional TNM stage classification method and the earlier published biomarkers on the prognosis of STAD patients.

**Conclusion:**

In this study, based on the early-stage specifically expressed genes, the prognostic signature constructed through TCGA and GEO datasets may become an indicator for clinical prognosis assessment of STAD and a new strategy for targeted therapy in the future.

**Supplementary Information:**

The online version contains supplementary material available at 10.1186/s40001-022-00827-4.

## Background

Gastric cancer (GC) is the fifth most common malignant tumor world widely, and it is also the fourth leading factor of death caused by cancer [1]. Gastric adenocarcinoma (STAD), which accounts for 95% of GC, is the most common histological type of gastrointestinal malignant tumor. The most effective treatment is radical surgery combined with chemotherapy, postoperative radiotherapy, and lymph node dissection in the early-stage of STAD [2]. Nonetheless, the curative effect on the advanced-stage STAD was still limited [3], and the 5-year overall survival rate of patients with advanced STAD was less than 10% [4]. It is a pity that 65% of patients were in the advanced stage of STAD when first diagnosed, and even nearly 85% of patients with STAD had lymph node metastasis at the time of diagnosis. Because the early symptoms of STAD are not obvious or asymptomatic, and so far, there are no diagnostic markers which have sensitive and specific effects on the early diagnosis of STAD. Consequently, there is an urgent need to discover effective prognostic signature of STAD and to develop new therapies and strategies.

The classification of STAD patients by next-generation sequencing was a novel approach, which rapidly identified the tumor characteristics, and we could design the most appropriate treatment strategy [5]. In the past few decades, with the development and application of the high-throughput sequencing technology, large-scale biological data have become an effective resource for researchers to search for probable cancer biomarkers. Numerous biomarkers have been identified through bioinformatics analysis [6, 7]. The predictive characteristics of some gene expressions had great significance in the application of clinical prognosis and identification of biomarkers. Studies showed that compared with the STAD patients without metastasis, the expression levels of ALOX12B and PACSIN1 were higher in patients with tumor metastasis, and the survival rate of patients with high expression was significantly lower than that of patients without metastasis [8]. These two genes may be potential biomarkers of metastasis and poor prognosis, which provide more information for follow-up comprehensive treatment and diagnosis of GC. Cox regression analysis demonstrated that EMCN/MUC15 combination had a good effect on the prediction of overall survival (OS) of STAD patients [9]. EMCN/MUC15-related genes were found associated with angiogenesis, mitosis and immunity in cancer-related processes, and may be potential prognostic markers of GC. The up-regulation of mRNA expression of ZNF860 was an independent prognostic indicator of RFS in I/II stage patients with STAD [10]. Ye et al. constructed a risk model involving 13 metabolism-based genes with the TCGA dataset to predict the survival of STAD patients [11]. Unfortunately, there is no research on the risk prediction model for patients with early-stage STAD.

In this study, the early-stage specifically genes of STAD were systematically analyzed through The Cancer Genome Atlas (TCGA) dataset using various packages in R, and their main biological functions and participating signal pathways were explored by the GO and KEGG enrichment analysis. The early-stage specific genes-based prognostic signature of STAD was established through the Cox risk regression method and verified by the Gene Expression Omnibus (GEO) dataset GSE84437. According to the risk score based on the prognostic signature, STAD patients were divided into the high-risk group and the low-risk group. The correlation between the prognostic signature and clinical characteristics of STAD patients was also investigated.

## Materials and methods

### Data collection

High-throughput sequencing gene expression data and clinical information of STAD patients were downloaded from the TCGA database, including 293 cases of STAD tissues and 28 cases of normal tissues. The TCGA-STAD dataset was used to analyze the differences in gene expression and as a training set to construct patient prognostic characteristics based on risk ratios (Table 1). The samples with missing clinical factors or missing survival follow-up information were removed. The other STAD dataset, GSE84437, was downloaded from NCBI GEO database. The sequencing information of this data set is based on the chip platform of GPL6947 (Illumina HumanHT-12 V3.0 Expression BeadChip), which contained 433 STAD samples. The GSE84437 dataset was used as a validation set to test the validity and accuracy of prognostic signature (Table 1).

**Table 1:** Clinicopathological characteristics of training and test sets for STAD patients in datasets

Clinical factors	TCGA (%)	GSE84437 (%)
Stage		
I	43 (14.7)	n.d
II	93 (31.7)	n.d
III	126 (43.0)	n.d
IV	31 (10.6)	n.d
T		
T1	16 (5.5)	11 (2.5)
T2	62 (21.2)	38 (8.8)
T3	140 (47.8)	92 (21.2)
T4	75 (25.6)	292 (67.4)
N		
N0	95 (32.4)	80 (18.5)
N1	74 (25.3)	188 (43.4)
N2	66 (22.5)	132 (30.5)
N3	58 (19.8)	33 (7.6)
M		
M0	273 (93.2)	n.d
M1	20 (6.8)	n.d
Age		
< = 60	99 (33.9)	194 (44.8)
> 60	193 (66.1)	239 (55.2)
Gender		
Female	115 (39.2)	137 (31.6)
Male	178 (60.8)	296 (68.4)

### Data processing

We extracted the clinical information of patients with STAD from the TCGA database. According to the STAD clinicopathological staging (I–IV stage) criteria of American Joint Commission (AJCC), 293 patients were divided into early stage (I–II) and advanced stage (III–IV), including 43 cases and 250 cases, respectively. The gene expression difference between the early-stage and the advanced-stage STAD tissues and 28 normal tissues, respectively, was analyzed using the R language limma package [12]. The false discovery rate (FDR) less than 0.05 and the relative expression value (log2 fold change |) >  = 1.5 were taken as the threshold of significant genetic difference. Volcano map, principal component analysis (PCA) dot map and heat map were drawn with ggplot2 package and pheatmap package to show the difference analysis results [13]. The early-stage specific DEGs of STAD were retrieved using the VennDiagram package in R [14].

### GO and KEGG functional enrichment analysis

Utilizing the noncentral hypergeometric distribution of the ClusterProfile and ggplot2 packages in R, based on Gene Ontology (GO) and Kyoto Encyclopedia of Gene and Genome (KEGG), the biological functions of genes specifically expressed in the early-stage of STAD were interpreted and enriched from the aspects of cellular composition (CC), molecular function (MF) and biological process (BP), as well as biological pathways, diseases and drugs [15].

### The prognostic multiple genes signature construction

With the Survival and Survminer packages in R, based on the clinical information of STAD patients in TCGA data set, the early-stage specific DEGs of STAD obtained in the previous step was analyzed by the univariate Cox proportional hazard regression analysis, and the statistically significant DEGs was screened out (*P* < 0.01). Then, multivariate COX proportional hazard regression analysis with two-way regression method was used to screen out the DEGs related to the prognosis of STAD patients. The overall survival time (OS) prediction multiple-gene signature of STAD patients was established, and the risk score (RS) was also calculated. The calculation formula of RS was

$$\mathrm{Risk score}=\sum\limits_{{i=1}}^{n}{Coef}_{i}* {x}_{i}.$$

According to the median RS value, the patients with STAD were divided into the high-risk group and low-risk group. Log-rank test was used to analyze the survival of the STAD patients by Kaplan–Meier method. The effectiveness and sensitivity of the prognostic signature were assessed by calculating the area (AUC) under the receiver operating characteristic (ROC) curve of STAD patients in the training set and verification set. According to the age, gender, lymph node metastasis, clinicopathological and histological stages of STAD patients, the correlation between the prognostic signature and the clinical factors was analyzed in the training set and verification set. A Nomogram was drawn to predict the prognosis of patients with STAD more conveniently and objectively.

Patients were divided into subgroups based on lymph node metastasis, clinicopathology, histological stage and age in the training and validation sets of STAD patients to analyze the relationship between prognostic signature and clinical factors. To further assess the superiority of the early prognostic features of STAD obtained in this study, the model was compared with four other previously published models (5-gene feature proposed by Chang et al. [16], 6-gene features proposed by Cho et al. [17], 3-gene feature proposed by Wu et al. [18], and 4-gene feature proposed by Wang et al. [19]) for predictive performance, including K-M survival analysis, ROC curve analysis, Harrell consistency index (C-index), and decision curve analysis (DCA).

### Gene set enrichment analysis

To elucidate the molecular mechanisms involved in the early prognostic features of gastric cancer, the R package “limma” was used to analyze differentially expressed genes between high- and low-risk groups classified based on prognostic features. The pathways and functions of differentially expressed genes enrichment were predicted by GSEA method. *P*-value < 0.05 for NOM was statistically significant. The results were visualized using the R package “clusterProfile”.

### Expression verification of the prognostic signature

The GC cell lines (AGS and MGC-803) used for the experiments were purchased from the Shanghai Institute of Cell Biology, Chinese Academy of Sciences. The GC cell lines were placed in Dulbecco's modified Eagle's medium (DMEM) containing 10% fetal bovine serum (FBS; HyClone, Logan, UT, USA), 100 U/ml penicillin and 100 mg/ml streptomycin. Invitrogen, Carlsbad, CA, USA) and incubated at 37 ℃ and 5% CO2 in an incubator. Total RNA of the cell lines was extracted using Trizol reagent (Invitrogen, CN). Then, 2 µg of RNA was taken for cDNA synthesis using Advantage RT-for-PCR Kit (Clontech), and the cell lines were incubated with HiScript^®^ II One Step qRT-PCR SYBR^®^ Green Kit (Takara, Japan) for qRT-PCR (Additional file 3: Table S1). GADPH was used as an internal control for mRNA and protein. Expression data were calculated using the 2^−ΔΔCt^ method. Immunohistochemistry-based protein expression profiles of prognostic gene signatures in normal gastric and STAD tissues were obtained using the online tool Human Protein Atlas (HPA, https://www.proteinatlas.org/). Protein was extracted with RIPA lysis buffer containing protease inhibitors and measured with a standard bovine serum albumin (BSA) kit. The extracted proteins were separated by electrophoresis using 10% sodium dodecyl sulfate polyacrylamide gel (SDS-PAGE) and then transferred to polyvinylidene difluoride (PVDF) membrane (Millipore Corporation, Billerica, MA, USA). The membranes were blocked in the 5% non-fat milk for one hour at the room temperature, and then incubated with the primary antibody (A15935 Abclonal, Wuhan, China; abs110490 absin China; GTX46121 GeneTex, Texas, USA; ab74030, ab67315, ab180166, ab88249, ab198895, ab202123 and ab73288 Abcam, Cambridge, UK), which was purchased from Abcam (Cambridge, UK), at 4 ℃ overnight and then washed with TBST solution (Boster, China). These membranes were then incubated with secondary antibodies. Finally, ECL chemiluminescence detection system is used for signal detection.

## Results

### Identification of early-stage specific DEGs of STAD

**Fig. 1:**
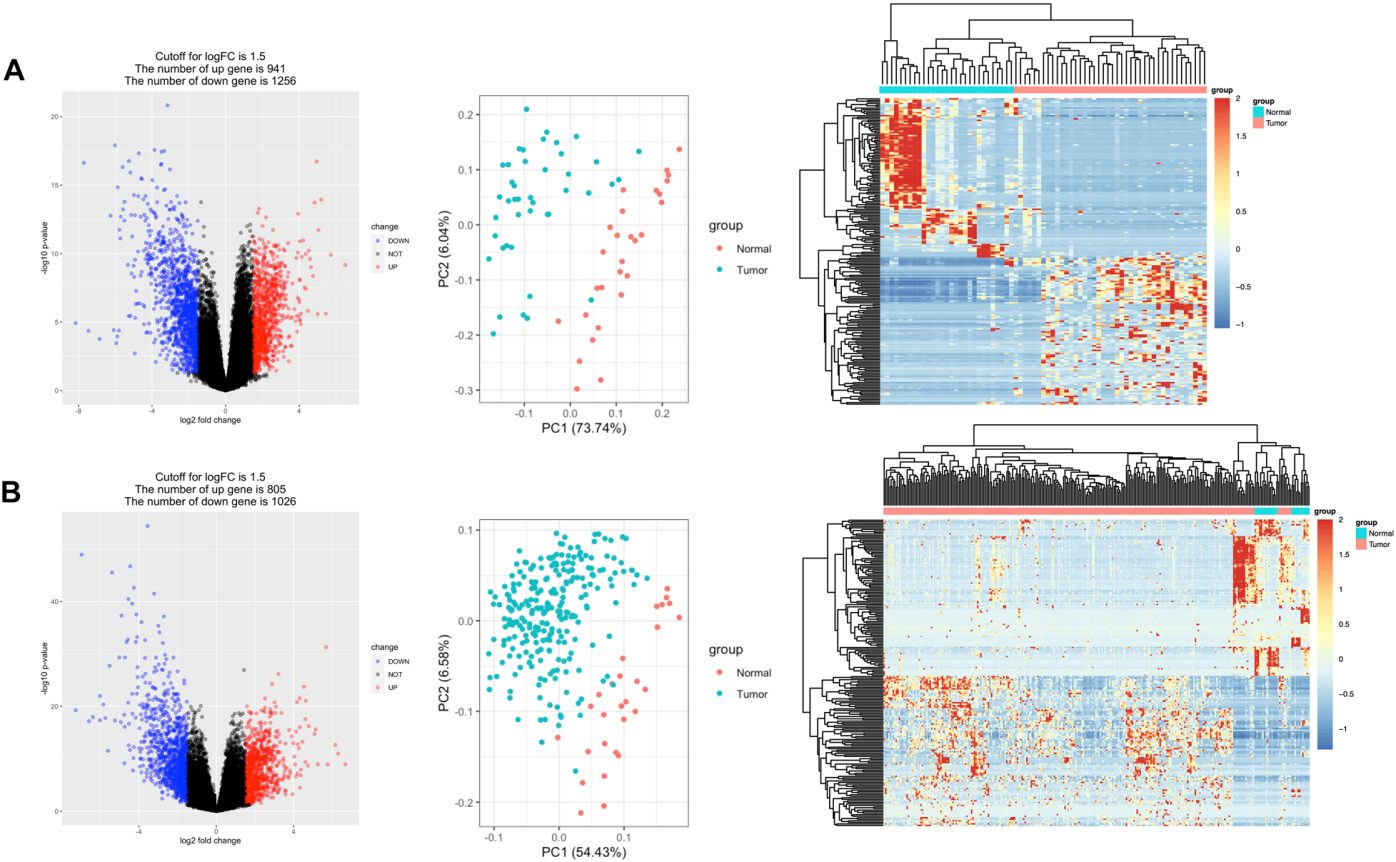
Volcano maps, PCA dot plots and hierarchical clustering heat maps of DEGs in TCGA-STAD datasets. X-axis: log2 FC; Y-axis: − log 10 (*p*-value) for each gene; vertical-dotted lines: | log2 FC |≥ 1.5; horizontal-dotted line: the significance cut off (*p*-value = 0.05). The red dot represents DEG. Expression heatmap of the top 100 DEGs between 43 cases of early stage STAD (**A**), 250 cases of advanced stage of STAD (**B**), and 28 cases of paracancerous normal tissues, respectively

**Fig. 2:**
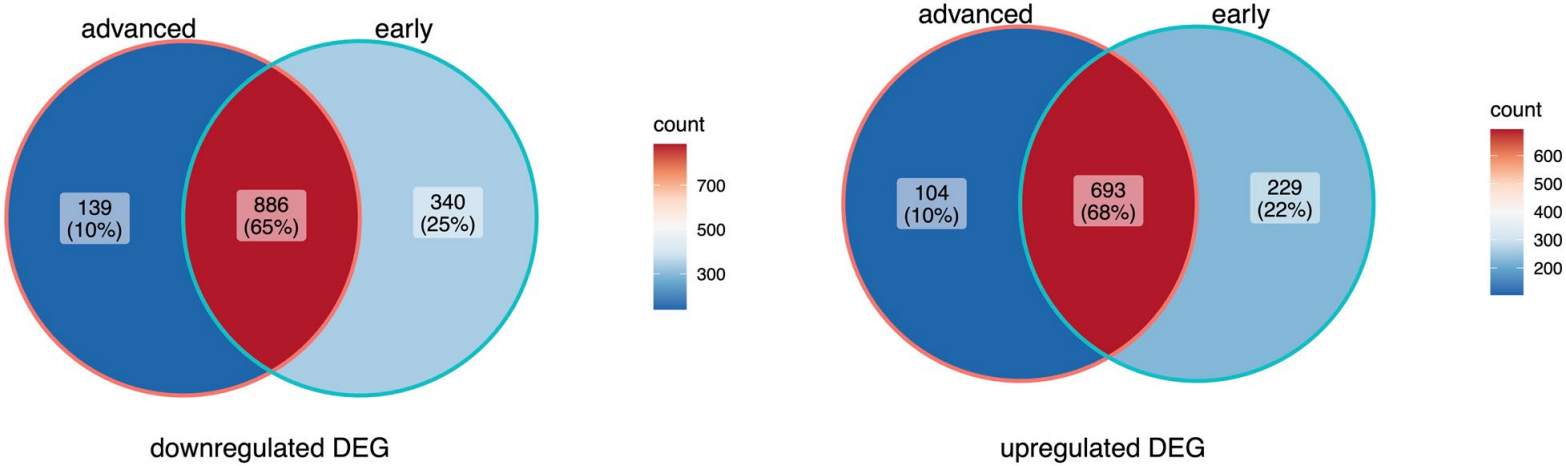
Venn diagrams of the overlapping DEGs in the advanced stage and early stage in TCGA-STAD datasets

Based on the high-throughput sequencing data in the TCGA-STAD dataset, the differential gene expression was analyzed between 43 cases of early-stage STAD tissues, 250 cases of advanced-stage STAD tissues and 28 cases of adjacent normal tissues, respectively. The results revealed that there were 2148 DEGs between early-stage STAD and normal tissues, including 992 up-regulated genes and 1226 down-regulated genes (Fig. 1A), and 1822 genes were differentially expressed between advanced-stage STAD and normal tissues, including 797 up-regulated genes and 1025 down-regulated genes (Fig. 1B). Based on the intersection analysis of these DEGs, we discovered that 886 DEGs and 693 DEGs overlapping in the early and advanced stages of STAD, respectively, as common down-regulated and up-regulated DEGs (Fig. 2). Notably, 569 DEGs were specifically expressed in the early-stage of STAD, including 340 down-regulated DEGs and 229 up-regulated DEGs. In addition, 139 down-regulated DEGs and 104 up-regulated DEGs were specifically expressed in the advanced-stage of STAD.

### Functional enrichment analysis of the early-stage specific DEGs

**Fig. 3:**
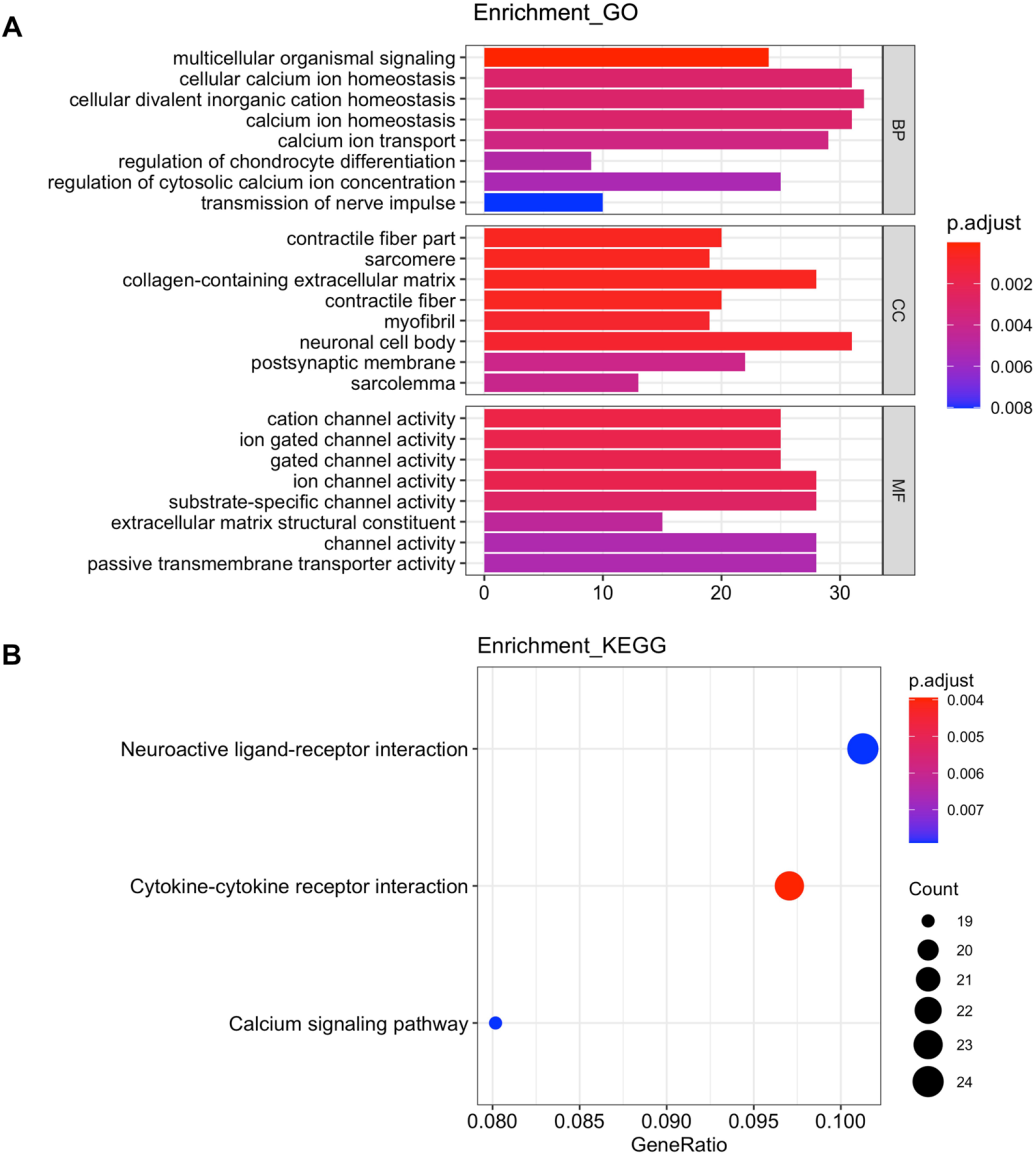
The significantly enriched in the GO categories, including BP, MF and CC (**A**), and KEGG pathways (**B**) of the early stage specific DEGs

Then, we performed GO and KEGG enrichment analysis to better study the function of the early-stage DEGs. GO analysis showed that these genes were mainly significantly enriched in multicellular organismal signaling (GO:0035637, *P* < 0.0001), cellular calcium ion homeostasis (GO:0006874, *P* < 0.0001), cellular divalent inorganic cation homeostasis (GO:0,072,503, *P* < 0.0001), contractile fiber part (GO:0044449, *P* < 0.0001), sarcomere (GO: 0030017, *P* < 0.0001), collagen-containing extracellular matrix (GO:0062023, *P* < 0.0001), cation channel activity (GO:0005261, *P* < 0.0001), gated channel activity (GO:0022836, *P* < 0.0001), and ion gated channel activity (GO:0022839, *P* < 0.0001, Fig. 3A, Additional file 4: Table S2). Meanwhile, KEGG pathway analysis revealed that the early-stage specific DEGs were mainly involved in pathways including cytokine–cytokine receptor interaction (hsa04060, *P* < 0.01), neuroactive ligand–receptor interaction (hsa04080, *P* < 0.01), and calcium signaling pathway (hsa04020, *P* < 0.01, Fig. 3B, Additional file 5: Table S3).

### Establishment of multigene Cox prognostic signature

Using the univariate Cox regression method, the 569 early-stage specific DEGs in the TCGA dataset were analyzed in combination with clinical parameters of STAD patients. Twenty-two genes that affect overall survival (OS) of STAD patients were screened with *P* < 0.01 as the threshold. Moreover, ten genes were identified as FERMT2, SLC52A3, TMTC1, GRP, AKAP12, GDF6, SLITRK4, NUDT11, RECK and MAGEH1 by two-way stepwise regression analysis, and the prognostic signature consisting of the ten genes were established. The expression of SLC52A3 in early-stage STAD tissues was significantly higher than that in normal tissues, while the expression of the other 9 DEGs in early-stage STAD tissues was significantly lower than that in normal tissues (*P* < 0.001, Table 2).

**Table 2:**
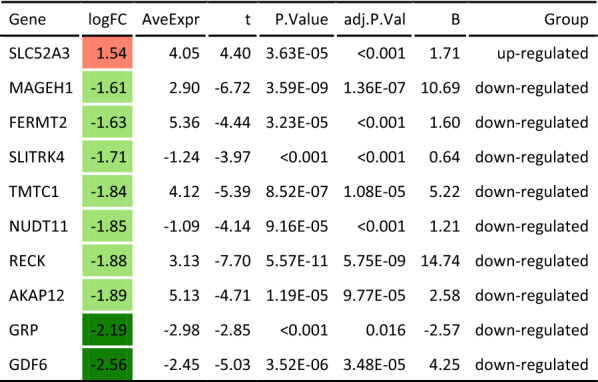
The DEGs in the prognostic signature between the early-stage STAD and normal tissues

Red color represents gene expressed up-regulated in tumor tissues compared to normal tissues; green color represents genes expressed down-regulated in tumor tissues, whereas light green indicates logFC values between − 2 and − 1, and dark green indicates logFC values less than − 2

**Fig. 4:**
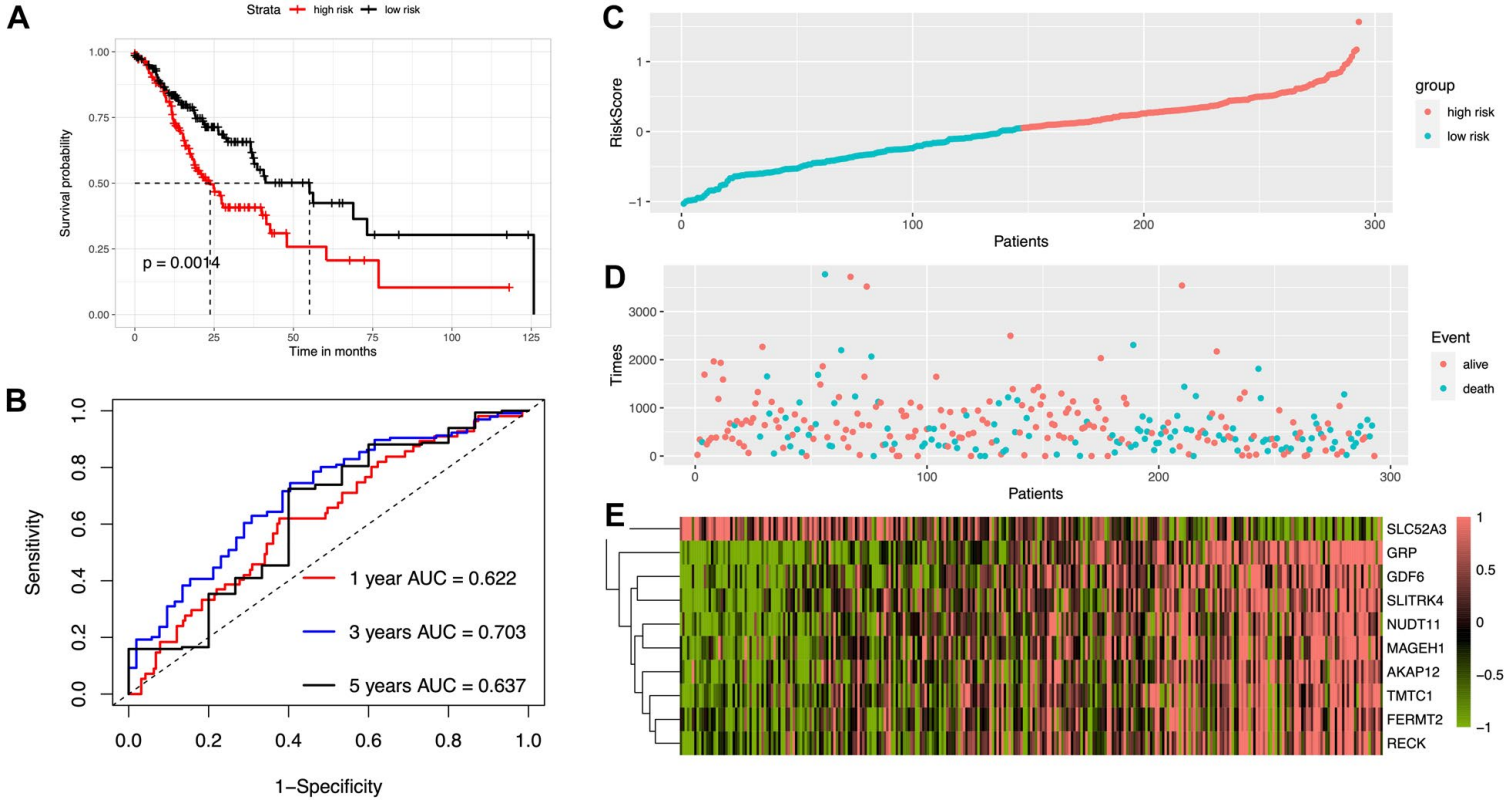
Construction of ten-mRNA signature from the early stage specific DEGs in the TCGA-STAD datasets as the training set. **A** KM survival analysis between the high- and low-risk groups in TCGA-STAD dataset. **B** Time-dependent ROC curves at 1, 3, and 5 years for patients’ OS of TCGA-STAD dataset. Relationship between survival status (**C**), survival time (days) (**D**) and risk score rank, respectively. The expression patterns of the ten genes in TCGA-STAD dataset (**E**)

Based on these 10 genes, the risk score formula was as follows: RS = − 0.308 × FERMT2 expression quantity − 0.166 × SLC52A3 expression quantity + 0.142 × TMTC1 expression quantity + 0.119 × GRP expression quantity − 0.004 × AKAP12 expression quantity + 0.034 × GDF6 expression quantity + 0.029 × SLITRK4 expression quantity + 0.079 × NUDT11 expression quantity + 0.008 × RECK expression quantity + 0.042 × MAGEH1 expression quantity. According to the median *RS* value (1.049) calculated by the prognostic score formula, 293 patients were divided into low-risk group and high-risk group. As shown in Fig. 4A, the survival rate in the low-risk group was significantly higher than that in the high-risk group (Log-rank *P* = 0.0014). The 1-year, 3-year and 5-year survival rates of the STAD patients were evaluated. The time-dependent ROC curve of the 10-gene signature showed that the AUC values were 0.622, 0.703 and 0.631, respectively, implying that the prognostic signature had a good prognostic ability (Fig. 4B). As shown in Fig. 4C, the higher the prognosis score, the more STAD patients died (Fig. 4D), indicating that the higher the risk score, the worse the prognosis of STAD patients. Figure 4E illustrates the gene expression of prognostic signature in the high-risk and low-risk groups.

**Fig. 5:**
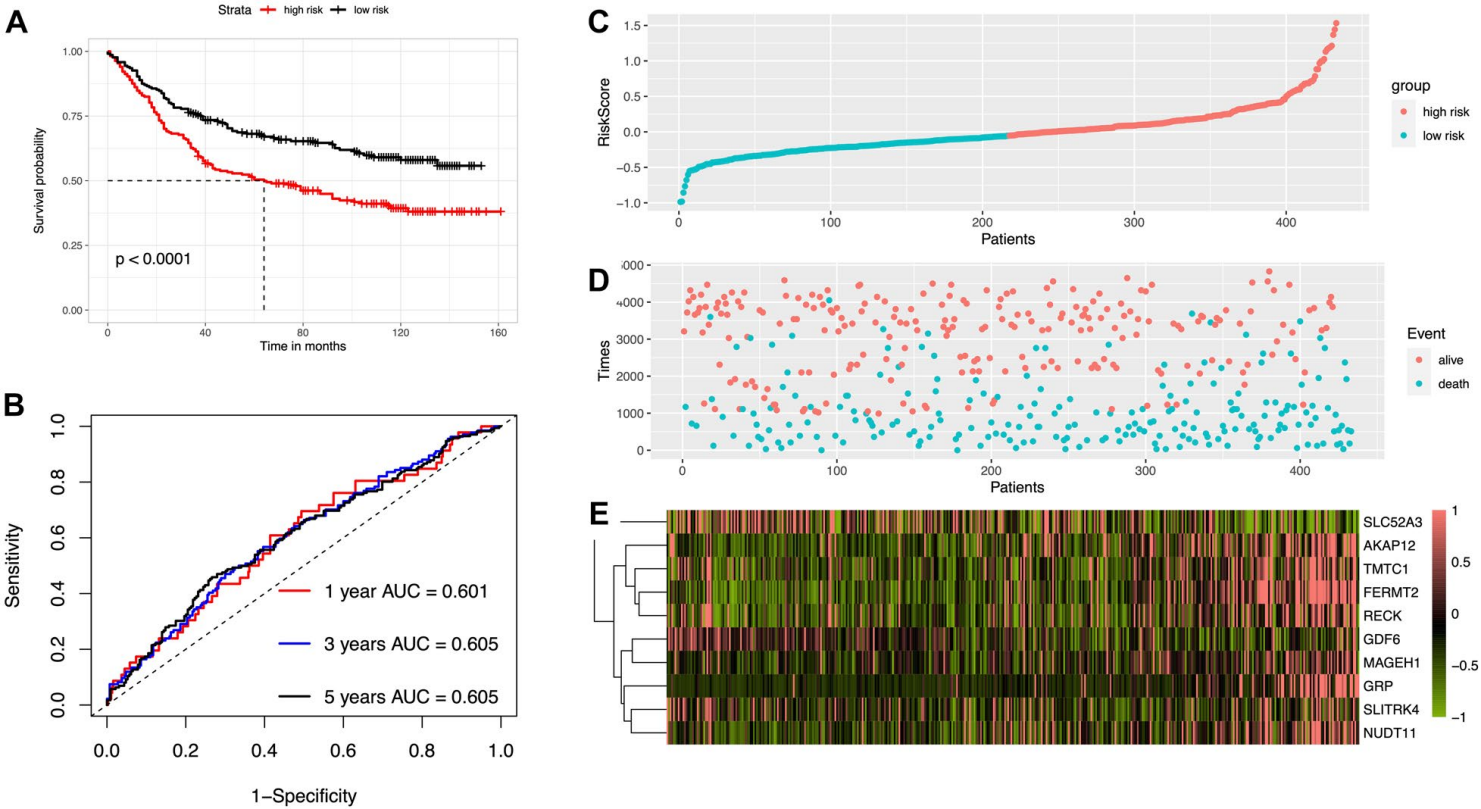
Validation of ten-mRNA signature from the early stage specific DEGs in the GSE84437 datasets. **A** KM survival analysis between the high- and low-risk groups in GSE84437 dataset. **B** Time-dependent ROC curves at 1, 3, and 5 years for patients’ OS of GSE84437 dataset. Relationship between survival status (**C**), survival time (days) (**D**) and risk score rank, respectively. The expression patterns of the ten genes in GSE84437 dataset (**E**)

Subsequently, the accuracy of the 10-gene prognostic signature was verified in the GSE84437 dataset. According to the median RS computed by the prognostic score formula (0.948), 433 STAD patients were divided into low-risk group and high-risk group. As shown in Fig. 5A, the OS in STAD patients was significantly higher in the low-risk group than in the high-risk group (*P* < 0.001). The 1-year, 3-year and 5-year survival of patients were analyzed, and the results indicated that the AUC values of the 10-gene prognostic signature were 0.601, 0.605 and 0.605, respectively, suggesting that the signature also had good prognostic ability (Fig. 5B). The higher the prognostic score (Fig. 5C), the more STAD patients died (Fig. 5D), demonstrating that the higher the risk score of STAD patients in the GSE84437 dataset, the worse the prognosis. Figure 4E shows gene expression in the high-risk and low-risk groups of polygenic prognostic markers. Furthermore, 1-, 3-, and 5-year OS in the STAD patients could be quantitatively predicted by the fraction of DEGs in the Nomogram (Additional file 1: Fig. S1).

### Correlation between the prognostic signature and clinical characteristics of STAD patients

Next, we analyzed the correlation between the prognostic signature and patients’ clinical characteristics. The results showed that in the TCGA-STAD dataset, there was a significant correlation between the high-risk group and the low-risk group in pathological stage, tumor size and patients’ survival status (*P* < 0.05), but there was no significant difference in lymph node, distant metastasis, age, and gender (Table 3). In the GSE84437 dataset, there was a significant correlation between the high-risk group and the low-risk group in tumor size and patients’ survival status, but there was no significant difference between the two groups in lymph nodes, age, and gender (Table 3). Taken together, the results showed that the 10-gene prognostic signature of STAD patients was related to the survival status, pathological stage, and tumor size.

**Table 3:** Correlation between prognostic model and clinical characteristics of STAD patients in TCGA and GSE84437 datasets

Factors	TCGA				GSE84437			
	High risk (*n* = 147)	Low risk (*n* = 146)	*χ*^2^	*P*	High risk (*n* = 217)	Low risk (*n* = 216)	*χ*^2^	*P*
Stage								
I	15	28	6.034	0.012	n.d	n.d	n.d	n.d
II	50	43			n.d	n.d		
III	63	63			n.d	n.d		
IV	19	12			n.d	n.d		
T								
T1	2	14	10.848	0.018	3	8	6.950	0.043
T2	31	31			14	24		
T3	76	64			43	49		
T4	38	37			157	135		
N								
N0	45	50	1.640	0.650	38	42	3.086	0.378
N1	35	39			91	97		
N2	34	32			74	58		
N3	33	25			14	19		
M								
M0	133	140	2.579	0.108	n.d	n.d	n.d	n.d
M1	14	6			n.d	n.d		
Age								
< = 60	65	76	1.649	0.199	101	104	0.056	0.811
> 60	82	69			116	112		
Gender								
Female	62	53	0.828	0.363	62	75	1.619	0.203
Male	85	93			155	141		
Event								
Alive	78	96	4.380	0.036	91	133	15.942	< 0.001
Dead	69	50			126	83		

We also compared the prognostic effects of the 10-gene prognostic signature and clinical factors on patients. The RS of 10-gene prognostic signature and clinical factors of patients in the TCGA-STAD dataset were included in the Cox regression analysis. RS of multivariate prognostic characteristics was a continuous variable, and clinical indicators of patients were sorted as classification variables. Univariate Cox regression analysis showed that pathological stage (*P* = 0.002), lymph node (*P* = 0.007), tumor size (*P* = 0.027), age (*P* = 0.004) and RS (*P* < 0.001) were risk factors affecting the OS in STAD patients (Table 4). Multivariate Cox regression analysis showed that the risk score of the prognostic signature (*P* < 0.001) was an independent factor influencing the prognosis of STAD patients (Table 4). Meanwhile, the RS of 10-gene prognostic signature and clinical factors in STAD dataset GSE84437 were also included in Cox regression analysis. Univariate Cox regression analysis showed that lymph node (*P* < 0.001), tumor size (*P* < 0.001), age (*P* = 0.003) and RS (*P* < 0.001) were also risk factors affecting OS in STAD patients (Table 5). Multivariate Cox regression analysis showed that lymph node (*P* < 0.001) and RS (*P* < 0.001) were still independent factors influencing the prognosis of STAD patients (Table 5). The results showed that the 10-gene prognostic signature was better than traditional clinical characteristics in prognostic prediction of patients with STAD.

**Table 4:** Factors associated with OS for STAD patients in TCGA datasets based on Cox proportional hazard analysis

Factors	Univariate Cox						Multivariate Cox					
	*b*	*SE*	*Wald χ*^2^	*P* value	HR	95% CI	*b*	*SE*	*Wald χ*^2^	*P* value	HR	95% CI
Model	0.72	0.14	4.98	< 0.001	2.06	1.55–2.73	0.77	0.15	5.12	< 0.001	2.17	1.61–2.91
Stage	0.35	0.11	3.08	0.002	1.41	1.13–1.76	0.25	0.19	1.3	0.195	1.28	0.88–1.86
Age	− 0.53	0.19	− 2.87	0.004	0.59	0.41–0.84	− 0.79	0.19	− 4.04	0.078	0.86	0.61–1.07
N	0.22	0.08	2.69	0.007	1.25	1.06–1.47	0.06	0.11	0.49	0.626	1.06	0.84–1.32
T	0.26	0.12	2.21	0.027	1.3	1.03–1.64	0.09	0.16	0.56	0.574	1.09	0.8–1.49
M	0.59	0.33	1.79	0.073	1.81	0.95–3.47						
Gender	0.32	0.2	1.63	0.104	1.38	0.94–2.04						
Grade	0.18	0.19	0.96	0.336	1.2	0.83–1.73

**Table 5:** Factors associated with OS for STAD patients in GSE84437 datasets based on Cox proportional hazard analysis

Factors	Univariate Cox						Multivariate Cox					
	*b*	*SE*	*Wald χ*^2^	*P* value	HR	95% CI	*b*	*SE*	*Wald χ*^2^	*P* value	HR	95% CI
Signature	0.62	0.11	5.87	< 0.001	1.86	1.51–2.29	0.58	0.11	5.23	< 0.001	1.79	1.44–2.23
N	0.52	0.08	6.33	< 0.001	1.68	1.43–1.97	0.43	0.08	5.16	< 0.001	1.54	1.31–1.82
T	0.55	0.12	4.65	< 0.001	1.74	1.38–2.2	0.43	0.12	3.46	0.121	1.04	0.81–1.67
Age	− 0.42	0.14	− 3.00	0.003	0.66	0.5–0.86	− 0.39	0.14	− 2.79	0.065	0.88	0.72–1.09
Gender	0.23	0.15	1.47	0.141	1.26	0.93–1.7						

## KM analysis in clinical subgroups

**Fig. 6:**
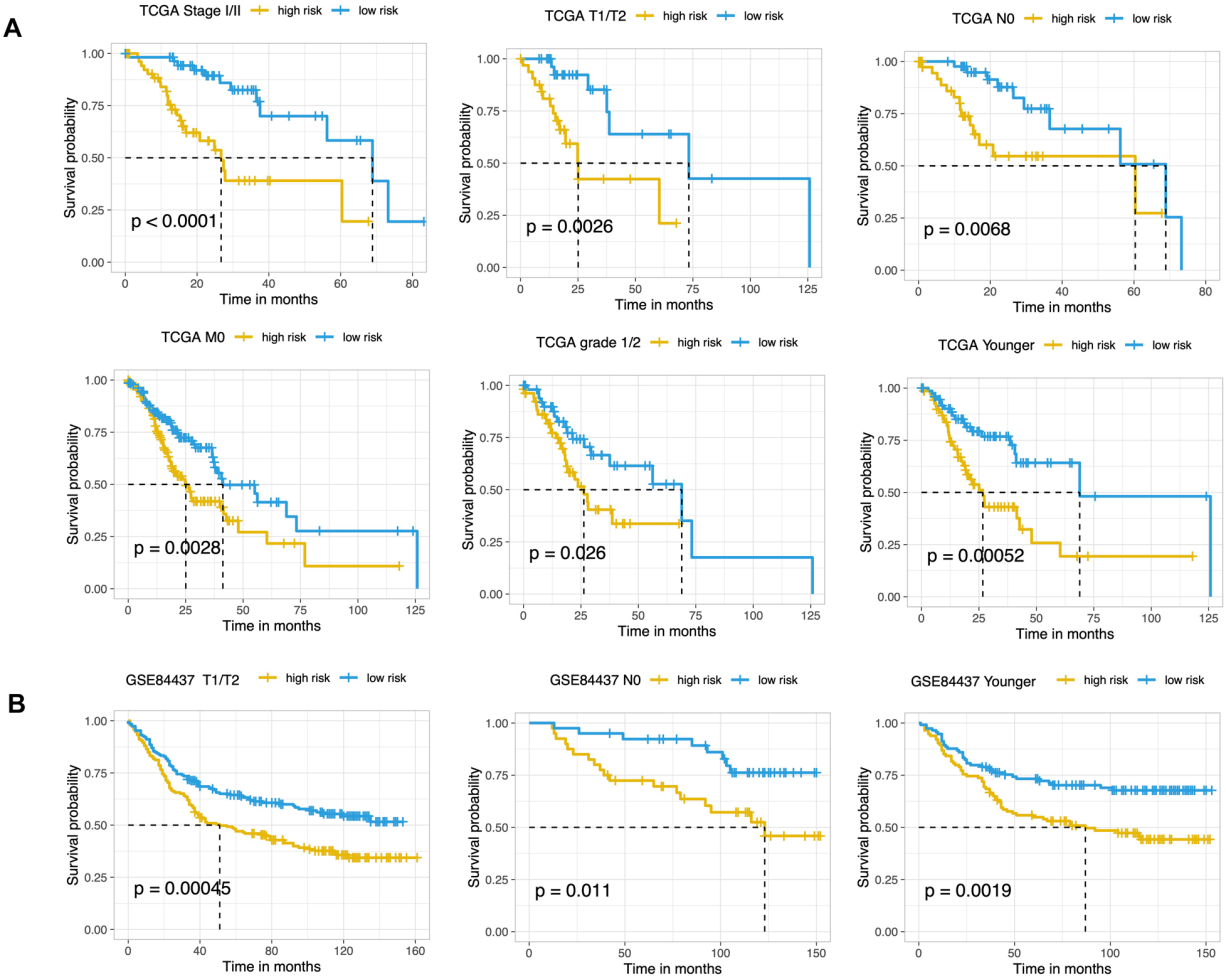
KM analysis of high- and low-risk groups in relation to the TCGA (**A**) and GSE84437 (**B**) dataset clinical subgroup

Furthermore, we investigated the relationship between 10-mRNA signature and overall survival of STAD patients in different clinical subgroups. It was found that in the TCGA-STAD dataset (Fig. 6A), clinical indicators such as non-lymph node metastasis stage (N0), clinical progression stage (Stage I to II), tumor grade T1–T2, early pathological grade (G1–G2), non-metastasis stage (M0) and younger age group, the OS was significantly better in the low-risk group with 10-mRNA signature than in the high-risk group. Similar results were revealed in GSE84437 dataset that patients with non-lymph node metastasis stage (N0), tumor grade T1–T2, and younger age group, the OS was also significantly higher in the low-risk group than in the high-risk group (Fig. 6B).

## Performance comparison of 10-mRNA signature and previous models

**Fig. 7:**
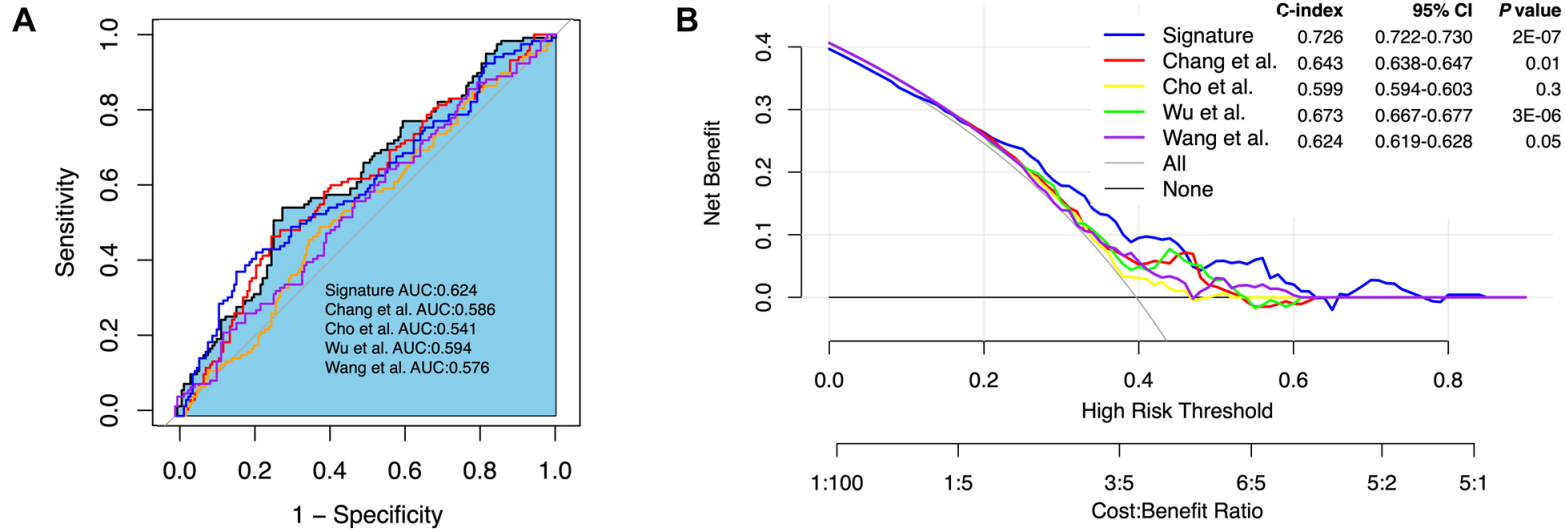
The performance of 10-mRNA signatures compared to previous signatures. ROC curves (**A**) and DCA profiles (**B**) in the TCGA-STAD dataset comparing the performance of 10-mRNA signatures compared to previous signatures, including Chang’s, Cho’s, Wu’s and Wang’s gene signatures

Several prognostic models for predicting survival in STAD patients have been reported in previous studies. We compared the predictive performance of the 10 gene signatures obtained in this study with four reported models. For normalization, the gene expression levels involved in each model were extracted uniformly from the original matrix of the TCGA-STAD dataset. Risk scores for STAD patients were calculated based on the corresponding coefficients for each model. Patients were included in the high-risk and low-risk groups according to the median risk score. We compared the ROC curves, CI values (95% CI values) and DCA curves, and the results showed that the 10-mRNA model established in this study had higher AUC values than the other signatures and the highest C-index among the five models (Fig. 7). This further demonstrated the better clinical utility of the 10-mRNA signature in predicting the survival of STAD patients.

### Expression validation

**Fig. 8:**
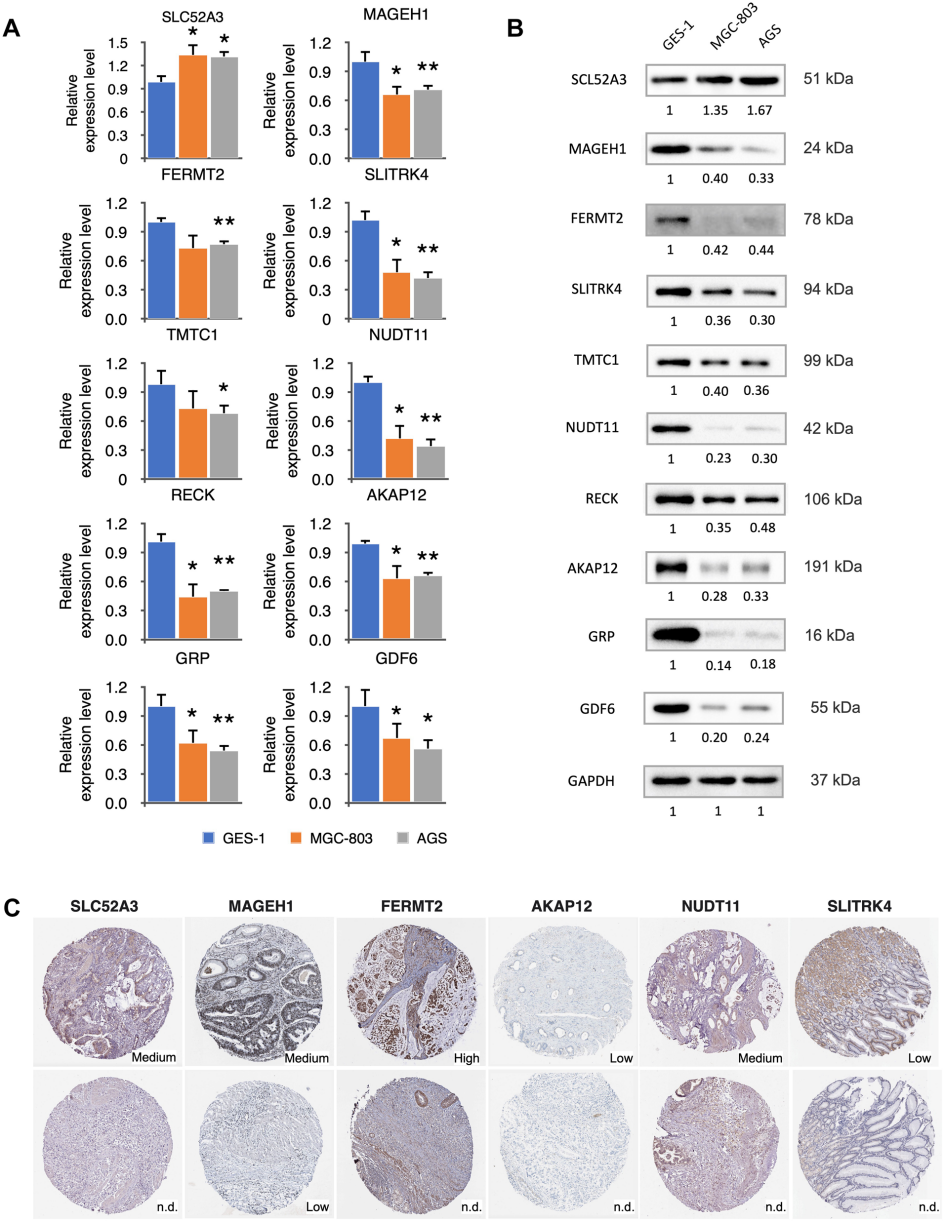
The expression of the signature genes in the human STAD cell lines (MGC-803 and AGS), human normal gastric epithelial cell line (GES-1) in mRNA level (**A**) and protein level (**B**, **C**), respectively. Immunohistochemistry map of the representative protein expression of the signature genes SLC52A3, MAGEH1, FERMT2, AKAP12, NUDT11, and SLITRK4 in STAD and normal gastric tissue with the antibody HPA049391, HPA011324, HPA040505, HPA006344, HPA057684, and HPA000431. Data were from the Human Protein Atlas online database. * indicates *P* < 0.05, ** indicates *P* < 0.01

Expression of the 10-mRNA signature was detected using GC cell lines through *q*RT-PCR. The results are shown in Fig. 8A. Except for TMTC1 and RECK in GC cell line MGC-803, which showed no significant difference in expression from normal cell lines, the mRNA levels of SLC52A3, MAGEH1, FERMT2, SLITRK4, NUDT11, AKAP12, GRP and GDF6 were significantly higher in both GC cell lines AGS and MGC-803 than in normal cell line. The protein expression levels show that SCL52A3 were up-regulated in the GC cell lines, meanwhile the other proteins were down-regulated compared to the normal cell line (Fig. 8B, Additional file 2: Fig. S2). We also obtained immunohistochemical results of the 10-mRNA signature from the HPA database to support the role in tumor tissues (Fig. 8C).

### GSEA

**Fig. 9:**
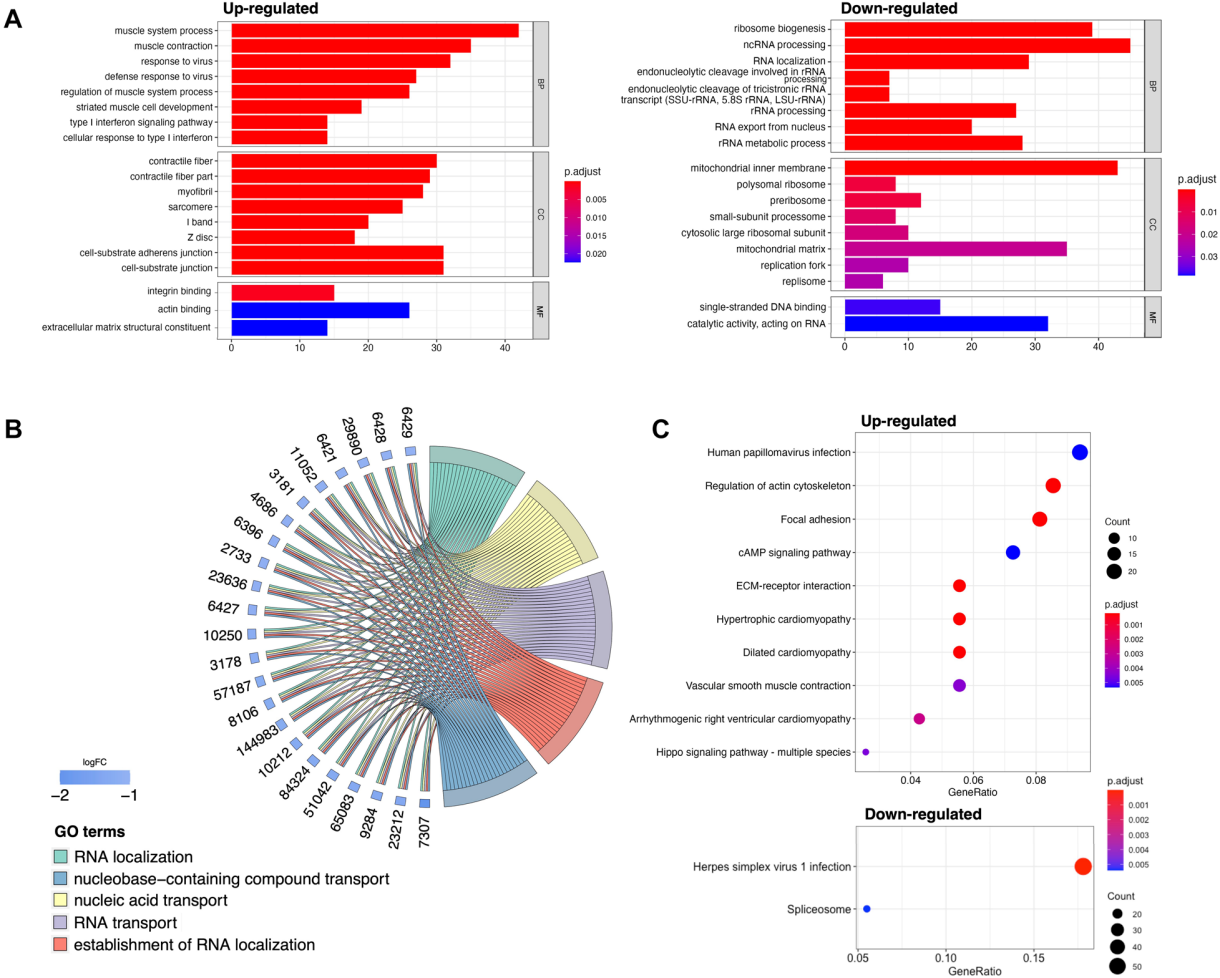
Functional enrichment and annotation analysis of DEGs between the high- and low-risk groups by the 10-mRNA signature. **A** The bar plots showing GO enrichment analysis; **B** the significant GO terms associated with the DEGs. **C** The dot plots showing KEGG pathway enrichment analysis. *p*-value less than 0.05 indicated a significant enrichment term

To explore the possible involvement of the 10-mRNA signature in tumor biological pathways, we performed differential gene expression analysis on the low- and high-risk groups classified according to the median RS value calculated by the prognostic scoring formula and obtained 521 expressed up-regulated genes and 872 expressed down-regulated genes. GSEA analysis of these DEGs revealed showed that significantly enriched activated and suppressed pathways, respectively (Fig. 9). Results indicate the up-regulated DEGs were enriched in GO terms including muscle system process (GO:0003012, *P* = 1.59E-09), contractile fiber (GO:0043292, *P* = 4.57E-11), integrin binding (GO:0005178, *P* = 0.001), etc., and pathways including ECM–receptor interaction (hsa04512, *P* = 0.0001), hypertrophic cardiomyopathy (hsa05410, *P* = 0.0001) (Additional file 6 and 7: Table S4, S5). Meanwhile, the down-regulated DEGs were enriched in GO terms including ribosome biogenesis (GO:0042254, *P* = 7.97E-08), mitochondrial inner membrane (GO:0005743, *P* = 0.001), single-stranded DNA binding (GO:0003697, *P* = 0.03), etc., and major pathways including Herpes simplex virus 1 infection (hsa05168, *P* = 6.52E-11), spliceosome (hsa03040, *P* = 0.005) (Additional file 6 and 7: Table S4, S5).

## Discussion

STAD is one of the most common malignant tumors of the digestive system, and its morbidity and mortality are among the highest among all cancers in the world. The genesis and development of STAD is a complex multi-stage process involving many genetic and epigenetic changes. The effectiveness and strategies of cancer therapy often depend on the stage of cancer diagnosed. The diagnosis of STAD is most usually in the advanced stage, which leads to difficult diagnosis, poor prognosis, and high mortality. Patients with early-stage STAD can be cured and have a good prognosis, but the early-stage diagnosis of STAD is very challenging because it is generally mild or asymptomatic. Molecular markers based on coding or non-coding genes have great potential in predicting the prognosis of cancers. The development of molecular markers that can effectively identify early-stage STAD and have good prognostic effect is crucial for the therapy strategy and effect of STAD.

In this study, the gene expression profiles of STAD samples and patients’ clinical factors were retrieved from TCGA and GEO public databases with bioinformatics methods. A total of 569 early-stage specific genes were identified by mining the differentially expressed genes in the early-stage of STAD. Enrichment analysis showed that these early-stage STAD specific DEGs were mainly involved in cytokine–cytokine receptor interaction, neuroactive ligand–receptor interaction, and calcium signal pathway. Then, univariate and multivariate Cox proportional hazard regression analysis of the early-stage specific DEGs was carried out by two-way stepwise regression, and 10 DEGs such as FERMT2, SLC52A3, TMTC1, GRP, AKAP12, GDF6, SLITRK4, NUDT11, RECK and MAGEH1 were obtained, and a prognostic signature based on these 10 DEGs was established. The survival analysis of TCGA-STAD test dataset and the GEO verification dataset GSE84437 revealed that the signature had a good predictive value for the prognosis of STAD patients in both datasets. Meanwhile, the time-dependent ROC curve analysis showed that the 10-gene signature had a good prediction effect. Through the analysis of the correlation between the 10-gene signature and the clinical characteristics of STAD patients, it was identified that this signature was related to the survival status, tumor pathological stage and tumor size of STAD patients. Cox regression and comparison analysis implied that the 10-gene signature was more efficient and sensitive than the traditional TNM stage and the earlier publishes biomarkers.

It was discovered that the expression of SLC52A3 in the early-stage STAD tissues was significantly higher than that in normal tissues, while the expression of the other 9 DEGS was significantly declined. Studies have found that FERMT2, SLC52A3, TMTC1, GRP, AKAP12, GDF6, SLITRK4, NUDT11, RECK and MAGEH1 genes are associated with tumor progression and prognosis. NF-κB p65/Rel-B can activate the expression of SLC52A3, and SLC52A3 was identified as a novel therapeutic target for esophageal cancer. The expression of FERMT2 was inhibited by miR-338-5p. Study has shown that FERMT2, which participated in cell proliferation and migration, and cisplatin resistance, performed as an oncogene in esophageal squamous cancer cells [20]. FERMT2 together with FKBP3, SMAD9, GATA2, and ITIH4 was constructed as a prognostic signature of lung cancer based on the differential expression of immune genes [21]. TMTC1 altered the proliferation and survival of cancer cells by participating in cell proliferation and inflammation, as well as the development of endoplasmic reticulum stress [22]. AKAP12 overexpression decreased hepatocellular carcinoma (HCC) cell proliferation, migration and invasion through targeting by miR-1251-5p [23]. The melanocyte differentiation gene MITF and the proapoptotic factor SOX9 were negatively regulated by GDF6, which blocked melanoma differentiation, inhibited cell death, and promoted tumor growth [24]. In HCC, SLITRK4 was inhibited by miR-139-5p, and the expression of SLITR4 played a role in cell invasion and proliferation [25]. The above reports implied the potential impact of the 10-gene prognostic signature constructed on STAD. The expression on mRNA and protein levels were also validated through GC cell lines and HPA databases. Because the STAD datasets in this study were obtained from TCGA and GEO databases, more clinical data samples need to be collected to verify the validity and reliability of the 10-gene prognostic signature. The biological function of genes in the signature was also worth investigating in further.

## Conclusion

By analyzing the gene expression difference between tumor and normal tissues in TCGA-STAD dataset, we obtained 569 early-stage specific DEG. KEGG and GO enrichment analysis gave us an insightful view of the functions of the early-stage specific DEGs. Subsequently, based on the TCGA-STAD and GSE84437 datasets, 10 early-specific mRNA prognostic signature was constructed, including FERMT2, SLC52A3, TMTC1, GRP, AKAP12, GDF6, SLITRK4, NUDT11, RECK, and MAGEH1, which were associated with tumor size and stage. The prognostic signature had a better effect than the traditional TNM staging method and previous published biomarkers in predicting the prognosis of STAD. In future, we will explore the prognostic genes in the signature and their potential function based on the current study.

## Supplementary Information

**Additional file 1: Fig. S1.** Nomogram of the prognostic signature for patients with STAD.

**Additional file 2: Fig. S2.** The original images of WB in this study to detect the protein expression level of 10-mRNA signature and GAPDH which as the internal control.

**Additional file 3: Table S1.** The sequences of primers used in this study.

**Additional file 4: Table S2.** GO terms enriched the genes differentially expressed associated with early-stage STAD.

**Additional file 5: Table S3.** KEGG pathways enriched the genes differentially expressed associated with early-stage STAD.

**Additional file 6: Table S4.** GO terms enriched the genes differentially expressed associated with the high-risk vs. low-risk group patients with STAD.

**Additional file 7: Table S5.** KEGG pathways enriched the genes differentially expressed associated with the high-risk vs. low-risk group patients with STAD.

## Data Availability

The TCGA and GEO belong to public databases. The patients involved in the database have obtained ethical approval. Users can download relevant data for free for research and publish relevant articles. Our study is based on open-source data, so there are no ethical issues and other conflicts of interest.

## Abbreviations

AUC: The area under the curve
BP: Biological processes
CC: Cellular components
DEGs: Differentially expressed mRNAs
FC: Fold change
GC: Gastric cancer
GEO: The gene expression omnibus
GO: Gene Ontology
HCC: Hepatocellular carcinoma
KEGG: Kyoto Encyclopedia of Genes and Genomes
m6A: N6-Methyladenosine
MF: Molecular functions
mRNAs: Messenger RNAs
K-M: Kaplan–Meier
OS: Overall survival
ROC: Receiver operating characteristic curve
RS: Risk score
TCGA: The cancer genome atlas
