# Supplementary material for: Construction of mRNA prognosis signature associated with differentially expressed genes in early stage of stomach adenocarcinomas based on TCGA and GEO datasets
Source: Eur J Med Res. 2022 Oct 17;27:205. doi: 10.1186/s40001-022-00827-4 (PMC9578190; doi:10.1186/s40001-022-00827-4)
Supplement: Supplementary file 5 — Additional file 5: Table S3. KEGG pathways enriched the genes differentially expressed associated with early-stage STAD. [file 40001_2022_827_MOESM5_ESM.docx]

**Table S3 The sequence of primers in this study**

| Name of primers | sequence |
| --- | --- |
| SLC52A3 F | 5’- ATG CAC CTG CTG GTC TGC GTC TT-3’ |
| SLC52A3 R | 5’- TGG CCA GCT GGA TGA CCA CCG T-3’ |
| MAGEH1 F | 5’-CCG TAA TGC GAG AGC CGC AGA AGA-3’ |
| MAGEH1 R | 5’-GGG CCG CTC AGG TCG TCT TC-3’ |
| FERMT2 F | 5’-ACC GCG ATG TCA CCC TGA GAG T-3’ |
| FERMT2 R | 5’-TTC AGA AGC CAA GTT CTC TTC TTT TCC-3’ |
| GADPH F | 5’-CTT CGC TTC GCT ATC ATC GAC G-3’ |
| GADPH R | 5’-TCG AAC GTC TAG CGA AGC TA-3’ |
| SLITRK4 F | 5’-CTG ATT TTG TCA GCC CTG ATT TCT TC-3’ |
| SLITRK4 R | 5’-ATT TGG TCT GTA GAC TGA AAC CTT CTC ACA-3’ |
| TMTC1 F | 5’-TGG TGA CCA CCT CTG CCC GAG-3’ |
| TMTC1 R | 5’-ATC GCC CAC ACG TCG TCG TGC A-3’ |
| NUDT11 F | 5’-CAA CCA GAC GCG GAC CTA CGA-3’ |
| NUDT11 R | 5’-CGG CAC GAT CCA GCG GTC-3’ |
| RECK F | 5’-TCC GGG CAG TGC GGG TGC AT-3’ |
| RECK R | 5’-TGG GGC TCG CTG CAA CAG ATG TTT-3’ |
| AKAP12 F | 5’-ATT GCC TGG GAT GCT GGC GCT-3’ |
| AKAP12 R | 5’-GGG TCA GCC CTC AGC TGC-3’ |
| GRP F | 5’-CTG GTC CTG CTG GCG CTG GT-3’ |
| GRP R | 5’-AGA AGA AGA CTC CCC TGT GCT CTT T-3’ |
| GDF6 F | 5’-CTC TCG GCC GTC TTC CTC ATC AG-3’ |
| GDF6 R | 5’-ATC TTG CCT TCC TTG CGG CTT CGC-3’ |
